# The Upper Common Pathway in Atrioventricular Nodal Reentrant Tachycardia: A Comprehensive Review of Evidence and Current Perspectives

**DOI:** 10.31083/j.rcm2503109

**Published:** 2024-03-15

**Authors:** Soyoon Park, Jeong-Wook Park, Young Choi

**Affiliations:** ^1^Division of Cardiology, Department of Internal Medicine, Seoul St. Mary’s Hospital, College of Medicine, The Catholic University of Korea, 06591 Seoul, Republic of Korea; ^2^Cardiovascular Research Institute for Intractable Disease, College of Medicine, The Catholic University of Korea, 06591 Seoul, Republic of Korea

**Keywords:** atrioventricular nodal reentrant tachycardia, upper common pathway, anatomy, atrioventricular node, nodoventricular bypass tract, nodofascicular bypass tract

## Abstract

Atrioventricular nodal reentrant tachycardia (AVNRT) is the most common form of paroxysmal supraventricular tachycardia, and its diagnostic and therapeutic approaches have been well-established. Traditionally, AVNRT is understood to be an intranodal reentry having two bystander pathways; the upper common pathway (UCP) which connects to the atrium and the lower common pathway which connects to the ventricle. However, the existence of the UCP remains a subject of ongoing debate. The assertion of the UCP’s presence is supported by electrophysiological evidence suggesting that the atrium is not essential for the perpetuation of AVNRT. Nonetheless, numerous anatomical studies have failed to identify any structure that could be conclusively designated as the UCP. The histological and electrophysiological characteristics of the slow and fast pathways, which are the core components of AVNRT, suggest the inclusion of atrial myocardium in the reentry circuit. While clear interpretation of these discrepancies remains elusive, potential explanations may be derived from existing evidence and recent research findings concerning the actual AVNRT circuit.

## 1. Introduction 

Atrioventricular nodal reentrant tachycardia (AVNRT) is the most common type of paroxysmal supraventricular tachycardia, with a prevalence of 22.5/10,000 persons [1]. It is now well established that the circuit of AVNRT consists of two major pathways connecting the atrium to the atrioventricular node (AVN); these are the fast pathway (FP) and the slow pathway (SP) [2, 3]. Previous studies have demonstrated that AVNRT can exhibit dissociation from either the ventricle or the atrium [4, 5, 6, 7]. This has led to the introduction of the concept that AVNRT has an intranodal reentrant circuit that is linked to the atrium through the upper common pathway (UCP) and to the His bundle through the lower common pathway (LCP) [8]. While the electrophysiological and anatomical evidence for the LCP is well-established, there has been limited evidence supporting the existence of the UCP [9, 10]. The diagnosis and treatment of AVNRT have seen remarkable advancements over the decades, now allowing for over 90% complete resolution of the disease through a safe, catheter-based ablation procedure [11]. However, controversy continues regarding the actual reentry circuit of AVNRTs; specifically, whether the reentry circuit is confined to specialized cardiomyocytes within the AVN or includes perinodal atrial working myocardium [12]. In this review, we aim to evaluate the previous literature concerning the presence of the UCP in AVNRT and further, we seek to offer a perspective on the actual circuitry of AVNRT by examining recent findings.

## 2. The Concept of UCP

The UCP refers to a singular pathway connecting atrioventricular (AV) nodal tissue with the atrium. This concept was introduced to account for various electrophysiological phenomena observed in AVNRTs. A particularly representative phenomenon suggesting the presence of the UCP is the ventriculoatrial (VA) block during AVNRT [13]. Such observations would be inexplicable if the AVNRT’s circuit was not electrically isolated from the atrium. Consequently, AVNRT is commonly understood as an intranodal reentry and has been schematically represented as being connected to the atrium via the hypothetical AV nodal tissue, in other words, the UCP [13]. Furthermore, the observation that the atrio-His (AH) interval during atrial pacing at AVNRT cycle length is longer than the AH interval during AVNRT has been interpreted as evidence suggesting the existence of the UCP, and the ΔAH value is thought to reflect the length of the UCP [14]. Miller *et al*. [14] investigated the prevalence of UCP in AVNRT in 1987, and described that a UCP was present in 29% of cases based on the difference in the AH interval or antegrade atrioventricular Wenckebach block during atrial pacing. However, despite the conceptual acknowledegment of the UCP, the precise locations of the proximal and distal junction of the SP and FP that constitute the reentry circuit within the AVN have not been definitively elucidated. Jackman demonstrated that the atrial insertions of the SP and FP are distinct, suggesting that the AVNRT circuit is not confined solely to the AV node [15]. In another theory, the UCP is hypothesized to be intra-atrial transitional cells connecting the atrial ends of the slow and fast pathways that conduct to the atrial myocardium [16]. If the UCP is absent, the AVNRT circuit could be perceived as being composed of the SP, FP, and connecting atrial tissue [17, 18]. Nonetheless, it remains challenging to explain all the electrophysiological phenomena that favor the presence of the UCP, as manifested in various case reports to date, with this circuit [6, 19, 20, 21].

## 3. Anatomical Consideration for the Connections between the AVN and the Atrium

Recent anatomical studies have shown the histological evidence of potential pathways inferred as the FP and SP, and their relationship with the adjacent anatomic structure, including the AVN [22]. The AVN is located within the apex of the inferior pyramidal space, insulated from the underlying crest of the ventricular septal myocardium by a fibrous plate. There are inferior extensions originating from the AVN, which typically descend along the posterior septal vestibules of the tricuspid and mitral annulus [23]. In general, the right inferior extension is dominant, and in some individuals, the left inferior extension can even be absent [22, 23]. This finding is consistent with the fact that most AVNRT is interrupted by ablation from the right inferior extension [24]. However, there can be significant variation in the extent or location of the AV nodal extensions, which might enable superior or left lateral type AVNRT [25, 26]. Because the AVN is positioned within the inferior pyramidal space, those extensions are overlaid by the vestibular atrial tissues, thereby connecting it to the atrial tissues or coronary sinus (CS) musculature at the end of the extension [22, 27]. From a functional perspective, since these extensions contain a component from the AVN, they can provide input as the SP. At the level of the apex of the triangle of Koch, the AVN receives another atrial input from the central part of the atrial septum, which can be right or leftward, and superficial or deep [23, 28]. This atrial input is the last connection that conducts to the AVN just prior to its insulation by the fibrous tissues to become the His bundle, and therefore, a short distance to the His bundle with a nature of working atrial cardiomyocytes enables fast conduction and thus can provide input as the FP [22]. Taken overall, the AVN commonly has three inputs, and these correspond to the well-known anatomical locations and histology of the FP and the SP, respectively. However, other than the AVN, distinct anatomical structure for a direct connection between the FP and SP has not been identified. In other words, there has been a lack of anatomical evidence to support the presence of the UCP in typical AVNRTs so far. For atypical, slow-slow AVNRTs, the right and left AV nodal inferior extensions could be directly connected through the CS musculature, which could hypothetically act as a UCP. In this case, a closed loop for AVNRT using both the right and left SPs could be formed without working atrial cardiomyocytes, and thus this may explain AVNRT cases with a retrograde atrial conduction block [29].

## 4. Evidence Supporting the Presence of UCP

The presence of UCP is supported by evidence that the atrium is not a necessary component to sustain AVNRTs. Various types of VA dissociation during AVNRT have been considered as the representative evidence proving the existence of UCP [7, 30, 31]. It has been reported that the Wenckebach HA block can occur during AVNRT, with an identical atrial activation sequence to that during 1:1 HA conduction [31]. This phenomenon could be explained by the intranodal location of the reentry circuit, and rate-dependent decremental property of the UCP. Morihisa *et al*. [30] demonstrated various patterns of VA block in 9 patients with AVNRTs. Of the 12 incidents of VA block, the type was: Wenckebach block in 7, 2:1 VA block in 4, and intermittent block in one. In the study, selective elimination of the SP conduction at the inferoparaseptal right atrium was effective in suppressing AVNRTs in all patients. The authors postulated that subatrial tissue linking the FP and SP forms the UCP in AVNRT, which explains the multiple atrial retrograde activation sequences and effective ablation sites distant from the AVN [30]. Sustained AVNRT with persistent VA dissociation has also been identified previously [13, 29, 32]. During the tachycardia, the H-H interval was constant despite the variations in the A-A interval, suggesting the circuit was electrically dissociated from the atrium [29]. The coexistence of atrial fibrillation (AF) with AVNRT as another instance of VA dissociation has been also reported [33]. The Wenckebach-type AV block during atrial pacing at a cycle length that was equal to or longer than that of AVNRT with one-to-one VA conduction, supports the presence of a UCP [15]. The discrepancy between the retrograde and anterograde conduction can be explained by the heterogeneous conduction property of the UCP in each direction [9]. Other evidence supporting the presence of UCP includes a rare phenomenon of spontaneous induction of AVNRT without an atrial echo beat which suggests that the atrium is not an essential part of reentry [34]. Depolarization of atrial tissue in the vicinity of the AVN without resetting AVNRT also indicates an intranodal location of the reentry circuit [20]. However, there is a possibility that a premature depolarization generates a delay in the SP which prevents its penetration into the tachycardia circuit. The evidence supporting the presence or absence of UCP is summarized in Table 1 (Ref. [15, 18, 20, 23, 30, 31, 33, 34, 35, 36, 37, 38, 39]).

**Table 1.** **Evidence supporting the presence or the absence of upper common pathway in AVNRTs**.

Presence of UCP	Absence of UCP
∙ Ventriculoatrial block during AVNRT [30, 31]	∙ Histologic findings that are against single electrical connection between the AVN and atrium [23]
∙ Coexistence of atrial fibrillation and AVNRT [33]	∙ Different atrial ends of the fast and slow pathway [18, 35]
∙ Wenckebach atrioventricular block during atrial pacing at a cycle length equal to the AVNRT [15]	∙ Orthodromic capture of atrial electrogram near the AVN by atrial overdrive pacing during AVNRT [36]
∙ Depolarization of atrial tissue surrounding the AVN without resetting AVNRT [20]	∙ Ability of a late premature atrial depolarization from coronary sinus to reset tachycardia, without affecting the fast pathway site [37]
∙ Spontaneous induction of AVNRT without an atrial echo beat [34]	∙ Successful ablation of AVNRT at the inferoseptal region of the right atrium, distant from the AVN [38, 39]

AVNRT, atrioventricular nodal reentrant tachycardia; UCP, upper common pathway; AVN, atrioventricular node.

## 5. Evidence Supporting the Absence of UCP

The primary evidence supporting the absence of UCP stems from anatomical studies that could not present histological evidence to corroborate intranodal reentry [23]. If the circuit of an AVNRT comprises both the FP and SP, and their atrial ends are not linked by electrically isolated tissue from the atrium, it is theoretically challenging to conceive the existence of a UCP [35]. This is further substantiated in electrophysiological studies where the earliest retrograde atrial activation sites during retrograde conduction over the FP and SP differ [18]. Heterogenous retrograde activation patterns over the FP have also been reported, which do not support the concept of anatomically discrete retrograde FP [28]. If the atrial myocardium is involved in the AVNRT circuit, a method to validate its existence would be to demonstrate the orthodromic capture of the atrium by a pacing maneuver during tachycardia. Satoh *et al*. [36] showed that burst atrial pacing during AVNRT could orthodromically capture the atrial electrogram near the His bundle potential in 5 of 7 patients. This result would not be anticipated in intranodal reentries having a UCP, and suggests that the atrium participates in the reentry circuit. Similarly, the ability of late-coupled atrial premature depolarization (APD) delivered at the timing of FP’s refractory period to reset AVNRT suggests the absence of a UCP. Yamabe *et al*. [40] analyzed the response to late APDs delivered at multiple sites near the Koch’s triangle in 18 patients with typical AVNRT. The late APDs (LAPD) delivered at the CS ostium were able to reset AVNRTs without affecting the retrograde His atrial electrogram, which demonstrated that the perinodal atrium extending from the His bundle region to the CS ostium would be an integral limb of the AVNRT circuit. In a recent study, the prevalence of UCPs was estimated using the late-coupled APDs from CS ostium in 126 patients with typical AVNRT [37]. The LAPD could reset the AVNRTs without affecting the earliest retrograde atrial activation site in 96% of patients (absence of UCP), and the presence of UCP was suggested in only 2.4% of patients. The ability to achieve an effective target within the septal atrial tissue, distant from the actual AVN site during AVNRT ablation, also serves as evidence opposing the concept of intranodal reentry [38, 39]. Keim *et al*. [41] conducted intrasurgical mapping of FP and SP and reported that the two pathways have separate atrial locations. Moreover, cooling at the intermediate site between slow and fast pathways did not disturb sustaining the tachycardia, which challenges the hypothesis that a specific transitional tissue, which is electrically isolated from the atrium and connects the slow and fast pathway, constitutes the AVNRT circuit.

## 6. UCPs in Orthodromic Reentrant Tachycardias Using Atypical Bypass Tracts

The presence of atypical bypass tracts that connect between the AVN and the ventricle was first described by Mahaim [42, 43]. The nodo-ventricular bypass tract (NVBT) has been reported to have a decremental property and can preexcitate the ventricle by anterograde conduction [44]. More recent studies have described the characteristics of orthodromic reentrant tachycardias using concealed nodofascicular bypass tracts (NFBT) or NVBTs, which were not well-known previously [45, 46, 47]. These nodoventricular (NV)/nodofascicular (NF) orthodromic reentrant tachycardias (NFORTs) have an infranodal circuit, and there is no doubt that they can be dissociated from the atrium and connected to the atrium via a pathway that is not participating in the tachycardia [48, 49]. NV/NFORTs mimic AVNRTs in morphology and electrophysiologic characteristics, which makes accurate differentiation often challenging [50, 51]. Also, the presence of concealed NVBT/NFBT as bystanders in AVNRT can make an echo beat when a AVNRT terminates by a VA block, and then the AVNRT can be reinitiated with a transient prolongation of the tachycardia cycle length [30]. This phenomenon cannot be accurately identified by current electrophysiologic techniques, and would lead to the incorrect interpretation of a VA block as if there were a UCP in AVNRT. Several criteria have been suggested to discriminate the NF/NVORTs from AVNRTs [50]. A His-refractory premature beat can indicate the presence of bypass tracts. However, it cannot necessarily exclude the possibility of concealed NV/NFBT, nor can it determine whether they participate in tachycardia [13]. The conventionally used maneuver of corrected post pacing interval (PPI) – total cycle length (TCL) of <110 ms and stimulus-atrial interval - VA interval of <85 ms are of limited valve in distinguishing between NF/NVORT from AVNRT, because PPI can be unexpectedly prolonged in NV/NFORTs due to the decremental property of the bypass tracts [52]. While there have been numerous case reports on AVNRT with VA block, the actual incidence of VA block in narrow QRS tachycardia is rare [9, 30]. It would be challenging to clearly differentiate whether this phenomenon arises entirely from AVNRT with an underlying UCP or due to the presence of concealed NF- or NVBTs. Nonetheless, the effective ablation site for NF- or NVBTs and the SP are similar [47]. Even without accurate differentiation between the two tachycardias, the ablation procedure would be successful in most patients.

## 7. Conclusions

The long-standing debate regarding the presence of UCP in AVNRT stems from the discrepancy between the anatomical/electrophysiological findings which suggest the circuit of AVNRT involves perinodal atrium, and rare phenomena during AVNRT that are not consistent with this circuit. However, as knowledge about the AVNRT circuit has accumulated, it has become evident that most AVNRTs do not exhibit behavior consistent with intranodal reentry. Cases that have previously demonstrated atrial dissociation during AVNRT can largely be explained without postulating the existence of UCP. In that regard, the presence of UCP would not be a general characteristic of AVNRTs. Further research into the actual circuit of tachycardias exhibiting characteristics of intranodal reentry will advance our understanding of AVNRT.
